# Relationship between the Temporal Changes in Positron-Emission-Tomography-Imaging-Based Textural Features and Pathologic Response and Survival in Esophageal Cancer Patients

**DOI:** 10.3389/fonc.2016.00072

**Published:** 2016-03-29

**Authors:** Stephen S. F. Yip, Thibaud P. Coroller, Nina N. Sanford, Harvey Mamon, Hugo J. W. L. Aerts, Ross I. Berbeco

**Affiliations:** ^1^Department of Radiation Oncology, Dana-Farber Cancer Institute, Brigham and Women’s Hospital, Harvard Medical School, Boston MA, USA; ^2^Department of Radiology, Dana-Farber Cancer Institute, Harvard Medical School, Boston MA, USA

**Keywords:** pathologic response, PET imaging, texture analysis, esophageal cancer, neoadjuvant therapy

## Abstract

**Purpose:**

Although change in standardized uptake value (SUV) measures and PET-based textural features during treatment have shown promise in tumor response prediction, it is unclear which quantitative measure is the most predictive. We compared the relationship between PET-based features and pathologic response and overall survival with the SUV measures in esophageal cancer.

**Methods:**

Fifty-four esophageal cancer patients received PET/CT scans before and after chemoradiotherapy. Of these, 45 patients underwent surgery and were classified into complete, partial, and non-responders to the preoperative chemoradiation. SUV_max_ and SUV_mean_, two cooccurrence matrix (Entropy and Homogeneity), two run-length matrix (RLM) (high-gray-run emphasis and Short-run high-gray-run emphasis), and two size-zone matrix (high-gray-zone emphasis and short-zone high-gray emphasis) textures were computed. The relationship between the relative difference of each measure at different treatment time points and the pathologic response and overall survival was assessed using the area under the receiver-operating-characteristic curve (AUC) and Kaplan–Meier statistics, respectively.

**Results:**

All Textures, except Homogeneity, were better related to pathologic response than SUV_max_ and SUV_mean_. Entropy was found to significantly distinguish non-responders from the complete (AUC = 0.79, *p* = 1.7 × 10^−4^) and partial (AUC = 0.71, *p* = 0.01) responders. Non-responders can also be significantly differentiated from partial and complete responders by the change in the run-length and size-zone matrix textures (AUC = 0.71–0.76, *p* ≤ 0.02). Homogeneity, SUV_max_, and SUV_mean_ failed to differentiate between any of the responders (AUC = 0.50–0.57, *p* ≥ 0.46). However, none of the measures were found to significantly distinguish between complete and partial responders with AUC <0.60 (*p* = 0.37). Median Entropy and RLM textures significantly discriminated patients with good and poor survival (log-rank *p* < 0.02), while all other textures and survival were poorly related (log-rank *p* > 0.25).

**Conclusion:**

For the patients studied, temporal changes in Entropy and all RLM were better correlated with pathological response and survival than the SUV measures. The hypothesis that these metrics can be used as clinical predictors of better patient outcomes will be tested in a larger patient dataset in the future.

## Introduction

Esophageal cancer is among the most aggressive gastrointestinal cancers with a high mortality rate (1, 2). Neoadjuvant chemotherapy and, or, radiotherapy are commonly used in combination with surgery to provide systemic and local control of the disease (3–5). Pathologic examinations of the surgical specimen have shown that preoperative chemoradiation alone can eradicate viable tumor cells in 10–29% of the patients (3, 6–9). Early prediction of the pathologic response allows physicians to identify which patients may or may not benefit from chemoradiotherapy, subsequently selecting an effective therapy for individual patients, while avoiding complications associated with ineffective or unnecessary treatment.

Non-invasive position emission tomography (PET) imaging with ^18^F-fluorodoxyglucose ([^18^F]FDG) is increasingly utilized for imaging of glucose metabolism for esophageal cancer diagnosis, staging, and monitoring disease progression (10–13). Due to its quantitative nature, standardized uptake value (SUV) measures, such as maximum and mean SUV, have been used to quantify tumor characteristics (14, 15). Furthermore, the reduction of SUV_max_ and SUV_mean_ between the longitudinal PET images has been shown to be significant predictors of tumor response to preoperative therapy and patients’ overall survival (16–23). However, SUV_max_ and SUV_mean_ fail to capture the heterogeneity in intratumoral [^18^F]FDG uptake resulting from the spatial variations in biological and genetic properties (24–26). Intratumoral heterogeneity is often found in cancer patients and has been shown to correlate with poor prognosis and treatment resistance (27–29). Accurate quantification of tumor heterogeneity may lead to more accurate prediction of treatment response (30, 31).

Textural features extracted from PET images through complex mathematical models of the spatial relationship between multiple voxels and their neighborhood have been proposed to describe the tumor heterogeneity (25, 26, 32, 33). For example, gray level cooccurrence matrix (GLCM) textures, such as Homogeneity and Entropy, measure the local relationship between two voxels (voxel pair) (34, 35). Tan et al. observed that [^18^F]FDG uptake became more homogeneous in the tumors that responded to preoperative chemoradiotherapy (22, 36). They used local GLCM textures to describe the evolution of the [^18^F]FDG uptake during therapy and found that the textures outperformed SUV measure in predicting the pathologic response (22).

Regional textures, such as those derived from the run-length matrix (RLM) and size-zone matrix (SZM), assess the spatial relationship of voxels beyond two neighboring voxels (37, 38). In a study by Yang et al., 20 patients with cervical cancer were classified into metabolic complete responders, partial responders, and new disease according to the PET images acquired 3 months after the concurrent chemoradiotherapy (39). They observed that the early changes (2–4 weeks) in the RLM and SZM textures during the therapy were more sensitive than SUV measures in detecting the metabolic tumor responders (39). Their results therefore suggested that the RLM and SZM textures may be a more significant prognostic indicator than the SUV measures.

Although changes in SUV measures and PET-based textural features during treatment have shown promise in tumor response prediction, it is unclear which quantitative measure is the most predictive. In this study, we evaluated the relationship between the change in PET-based texture features and overall survival and pathologic response to preoperative chemoradiotherapy in esophageal cancer patients and compared to the same correlations of standard SUV measures. We attempted to generate a hypothesis regarding which texture features, if any, should be explored as predictors of pathologic response and patient outcome. Two sensitivity studies were also conducted to investigate the impact of change in PET resampling scheme and tumor volume on the association between textures and pathologic response.

## Materials and Methods

### Patient Characteristics and Imaging

This retrospective study was conducted under a Dana-Farber/Harvard Cancer Center institutional review board (IRB) approved protocol. All images and clinical data were analyzed anonymously and retrospectively. This study included 54 patients (10 females and 44 males, median age = 65 years) with esophageal cancer (one tumor/patient) received [^18^F]FDG–PET/CT scans, on average, 9 ± 1 weeks before and 5 ± 5 weeks after the chemoradiotherapy between August 2009 and April 2013. There were fifty patients with adenocarcinoma and four patients with squamous cell carcinoma. Table 1 shows the clinical tumor stage assessed before the initiation of treatment according to the TNM staging criteria established by the American Joint Committee on Cancer (seventh edition).

**Table 1:** **Patient characteristics**.

	No. of patients
**Prechemoradiotherapy staging (cTNM)**	
T-stage (T1/T2/T3)	2/16/36
N-stage (N0/N1/N2/N3)	16/23/14/1
M-stage (M0/M1)	53/1
**Postsurgery pathologic staging (ypTNM)^a^**	
T-stage (T0/T1a/T1b/T2/T3)	8/5/5/8/19
N-stage (N0/N1/N2/N3)	29/9/3/5
M-stage (M0/M1)	44/1

*TNM classification before and after chemoradiotherapy*.

*^a^Only 83% (45/54) of patients underwent surgery*.

Patients were injected with 10.3–22.4 mCi of [^18^F]FDG and scanned about 65 min after injection either on a GE scanner (GE Healthcare, Waukesha, WI, USA) or a Siemens Biograph PET/CT scanner (Siemens AG, Erlangen, Germany) based on the availability of the scanners. The acquisition time was 3–5 min/bed position for a whole-body scan from the base of skull to the proximal femora. The acquired PET data were reconstructed using 3D iterative VUE Point reconstruction (2 iterations/35 subsets for GE-DST) and order subset expectation maximization (2 iterations/28 subsets for GE-DSTE or DLS, 2 iterations/21 subsets for GE-DRX, and 4 iterations/8 subsets for Siemens Biograph). Attenuation correction of PET images was performed using the CT images. The types of crystals and the PET spatial resolution for each PET resolution are summarized in Table 2.

**Table 2:** **Types of crystals used and the PET spatial resolution for each PET/CT scanner**.

PET/CT scanners	Crystal type	PET spatial resolution
GE discovery ST	BGO	4.69 mm × 4.69 mm × 3.27 mm
GE discovery STE	BGO	4.69 mm × 4.69 mm × 3.27 mm
GE discovery RX	LYSO	4.69 mm × 4.69 mm × 3.27 mm
GE discovery LS	BGO	4.69 mm × 4.69 mm × 3.27 mm
Siemens biograph	LSO	4.06 mm × 4.06 mm × 5.00 mm

### Chemoradiotherapy

Total radiation dose of 45–50.4 Gy were delivered to the patients over 5 weeks with five fractions per week (1.8 Gy/fractions and 1 fractions/day). The concurrent chemotherapy received by 18 patients included cisplatin combined with 5-fluorouracil (5-FU), irinotecan, or paclitaxel. Thirty-six patients received a chemotherapy regimen consisting of carboplatin and paclitaxel.

### Surgery and Pathologic Response Classification

Of 54 patients, 45 (83%) underwent surgery after the concurrent chemoradiotherapy. Patients with low tolerance of surgery due to toxicity from chemoradiation and other medical problems were excluded from surgery. Surgery was performed, on average, 7 ± 2 weeks after preoperative chemoradiotherapy. All surgical specimens were examined and staged (ypTNM) by the pathologists (Table 1). Patients were further classified into complete responders, partial responders, and non-responders to preoperative chemoradiation. A complete responder was defined as having no microscopic evidence of viable tumor cells. A partial response was defined as the downstaging of pretreatment TNM staging. Patients who had no change or increased in tumor stage were defined as non-responders. Eight, twenty-two, and fifteen patients were identified as complete, partial, and non-responders, respectively.

### Textural Features

A large number of textural features computed from complex mathematical models of the spatial relationship among multiple image voxels can be extracted from medical images (25, 26, 32, 35, 40, 41). However, if we were to assess the ability of numerous textures in predicting pathologic response, then at least some textures would be shown to be predictive merely based on random chance alone (42).

Therefore, only six textures, including GLCM-derived Homogeneity and Entropy (34, 35) were assessed. RLM-derived high-gray-run emphasis and Short-run high-gray-run emphasis (37) and SZM-derived high-gray-zone emphasis and short-zone high-gray emphasis were included for the analysis (38). These six PET-based textures were chosen due to their potential clinical value in prognosis and treatment response assessment (22, 30, 31, 39).

### PET Textural Features Computation

We extracted the textural features from both the PET images acquired before (pretreatment) and after (posttreatment) chemoradiotherapy. Fifty-four tumor volumes were manually delineated by an experienced radiation oncologist using both the PET and CT images. For each patient, the CT counterparts of the pre- and posttreatment PET/CT images were deformably registered (43). The transformation resulting from the deformable registration was then applied to propagate the manually defined tumor volume on the pretreatment PET onto the posttreatment PET. The propagated tumor volumes were used to define the tumor region on the posttreatment PET. The deformable registration-based contour propagation has been shown to expedite the tumor contouring and texture quantification processes while not compromising the predictive ability of the textures (44).

Prior to texture computation, all PET images [PET($\vec{x}$)] were cropped to the tumor regions and processed using the following equation,

(1) $${\mathrm{PET}}^{\prime }(\vec{x})=2^{\text{N}}\cdot \frac{\mathrm{PET}(\vec{x})-\mathrm{minPET}}{\mathrm{maxPET}-\mathrm{minPET}}$$

where minPET and maxPET are the maximum and minimum intensities of PET within the tumor region. The intensity range of the postprocessed image $\left[{\mathrm{PET}}^{\prime }(\vec{x})\right]$ was resampled (or converted) into 256 (2^8^) discrete values.

We calculated the metabolic tumor volumes (MTV) as thresholded PET images with SUV over 40% of the maximum SUV within the tumor regions (45, 46). Within the MTV, the textural features were then computed using the MATLAB-based (The Mathworks Inc., Natick, MA, USA) Chang-Gung Image Texture Analysis Toolbox (47, 48). The maximum and mean SUV were also computed from the pre- and posttreatment PET images.

### Temporal Change in Quantitative Measures

The relative difference (ΔTexture) of each texture at different treatment time points was used to quantify the change in tumor texture values before and after chemoradiotherapy. The relative difference (ΔTexture) was defined as follows:

(2) $$\Delta \text{Texture}=\text{100\%}\cdot \frac{{\text{Texture}}_{\text{post}}-{\text{Texture}}_{\text{pre}}}{{\text{Texture}}_{\text{pre}}}$$

where Texture_pre_ and Texture_post_ are the textural features extracted from the PET images acquired before and after the chemoradiotherapy, respectively. We also defined ΔSUV to determine the change in the SUV measures, including SUV_max_ and SUV_mean_, before and after chemoradiation.

### Quantification of the ΔTexture and Pathologic Response Relation

Univariate analysis was performed with R (version 3.2) using the survcomp and pROC packages from Bioconductor (49, 50). We assessed the relationship between the quantitative measures and pathologic response by evaluating the performance of the measures to differentiate patients into different response classifications, including (1) complete from non-responders, (2) partial from non-responders, and (3) complete from partial responders. The performance was quantified using the area under the receiver operating characteristic curve (AUC). AUC is interpreted as the probability of correctly classifying the patients into different response categories (i.e., complete, partial, and non-responders). AUC ranges from 0 to 1 with the value 1 indicates perfect classification.

### Survival Analysis

Two analyses were performed to assess the relationship between the quantitative measures and patients’ overall survival. In the first analysis, the median value of ΔTextures or ΔSUV was used to stratify all 54 patients into two risk groups. Kaplan–Meier curves with log-rank statistics were then performed to compare the outcomes between these two groups. Unlike Kaplan–Meier analysis, concordance index (*c*-index) does not rely on a single cutoff value (i.e., median ΔTexture or ΔSUV). In the second analysis, we computed the *c*-index. Given two randomly drawn samples (patients), *c*-index determines the probability that an event (death) will happen to the sample with higher risk value (e.g., ΔTexture) (51, 52).

In our analysis, *c*-index and AUC <0.50 indicate that the ΔTexture (or ΔSUV) performs worse than random guessing (52–54). Noether test was used to determine if AUC or *c*-index was significantly greater than 0.50 with *p* < 0.05 for each quantitative measure (55, 56).

### Sensitivity Studies

We conducted two sensitivity studies to investigate the impact of change in PET resampling scheme and MTV on the performance of ΔTexture in differentiating pathologic response. In the first sensitivity study, PET images were also resampled to 32 (25), 64 (26), and 128 (2^7^) discrete values using Eq. (1). In the second study, PET-based textures were determined within the MTV as thresholded PET images with SUV over 30% (MTV_30%_), 50% (MTV_50%_), and 60% (MTV_60%_) the SUV_max_. The *default* parameters for the PET-based textures computation were 256 resampled discrete values and 40% SUV_max_ (MTV_40%_) threshold value.

## Results

### The Relationship between Pathologic Response and ΔTexture (ΔSUV)

**Figure 1:**
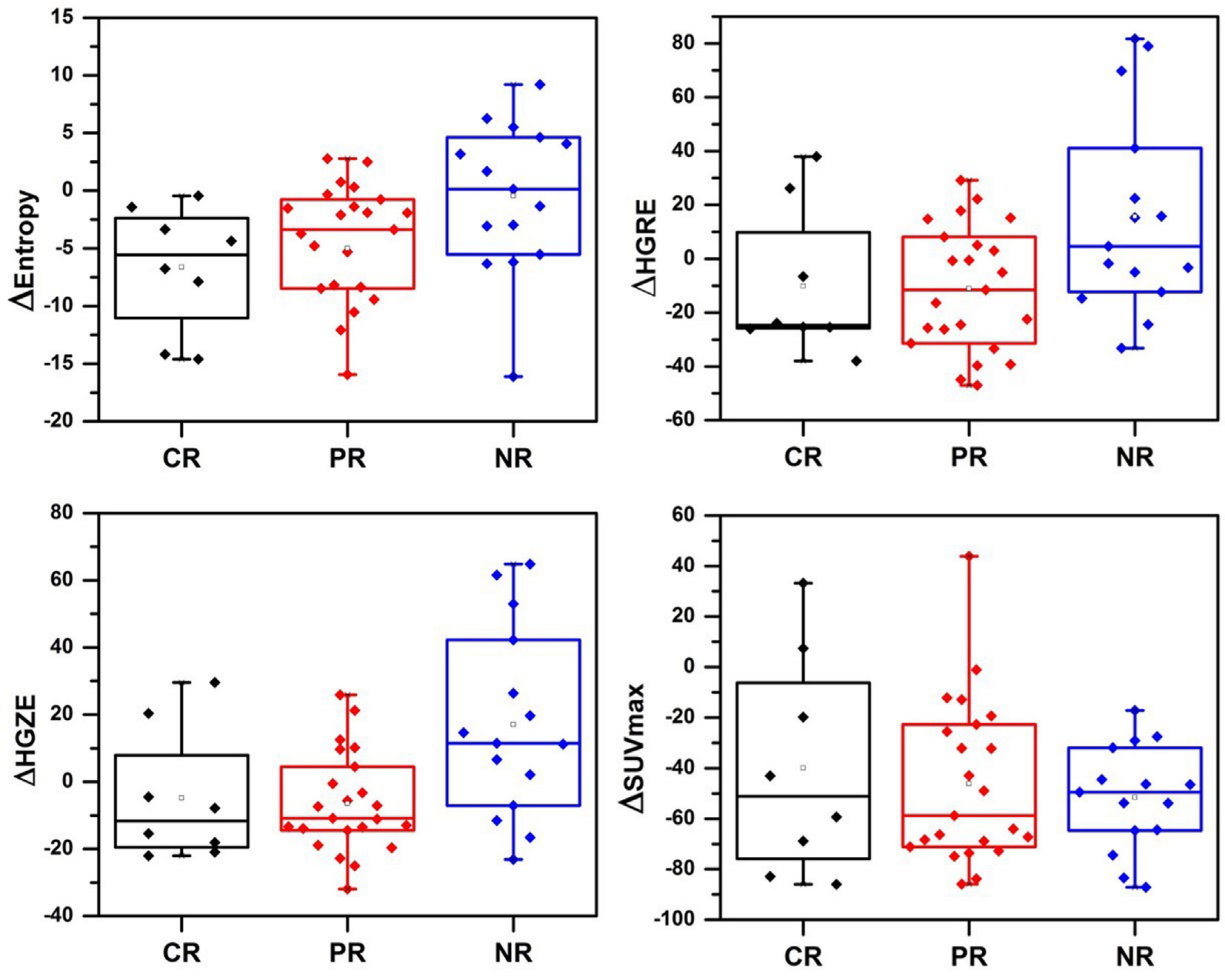
**Change in textures and SUV_max_ before and after chemoradiation**. CR, complete responders; PR, partial responders; NR, non-responder; HGRE, high-gray-run emphasis; HGZE, high-gray zone-run emphasis.

**Figure 2:**
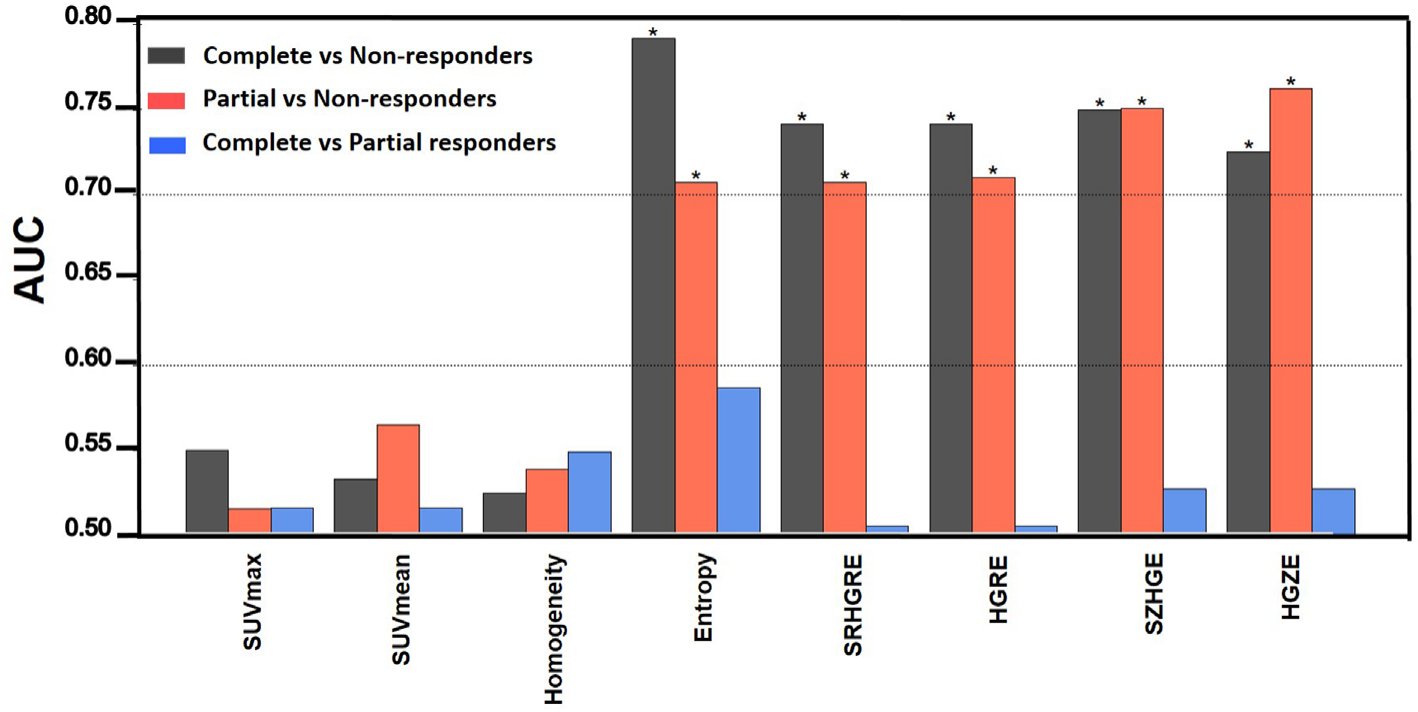
**The area under the receiver operating curve (AUC)**. * indicates *p* < 0.05. HGRE, high-gray-run emphasis; HGZE, high-gray zone-run emphasis; SRHGRE, Short-run high-gray-run emphasis; SZHGE, short-zone high-gray-run emphasis.

The boxplots in Figure 1 visually highlight the performance of four example measures in differentiating non-responders from complete and partial responders. The temporal changes in texture (ΔTexture) generally were observed to be better related to pathologic response than ΔSUV (Figure 2). ΔEntropy was found to significantly distinguish non-responders from the complete (AUC = 0.79, *p* = 1.7 × 10^−4^) and partial (AUC = 0.71, *p* = 0.01) responders. Non-responders can also be significantly differentiated from partial and complete responders by the change in the run length and SZM textures (AUC = 0.71–0.76, *p* = 7.7 × 10^−4^–0.02) (Figure 2). ΔHomogeneity, ΔSUV_max_, and ΔSUV_mean_ failed to separate any of the responders (AUC = 0.50–0.57, *p* > 0.46). However, none of the measures were found to significantly distinguish between complete and partial responders with AUC = 0.51–0.59 (*p* > 0.37).

### Sensitivity Studies

**Figure 3:**
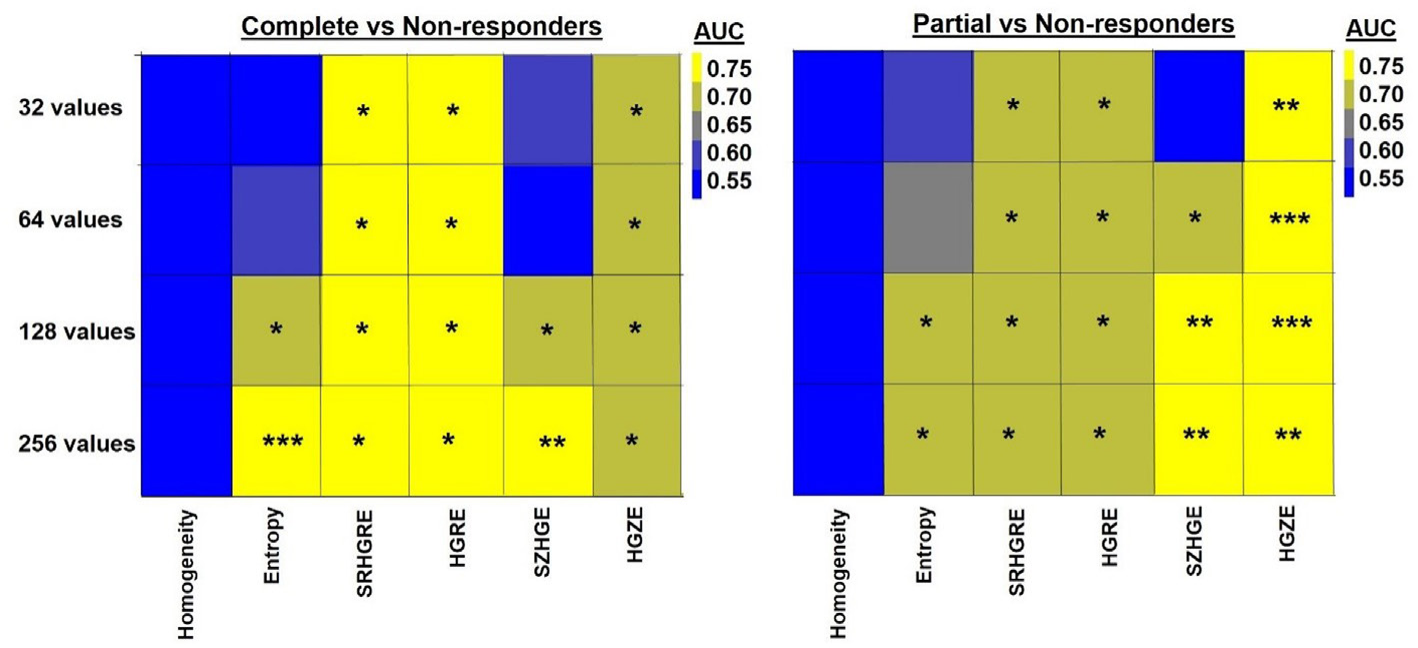
**Heatmap shows the quantification (AUC) of the relationship between ΔTexture and pathologic response computed with 32, 64, 128, and 256 discrete values**. * indicates 0.005 < *p* < 0.05, ** indicates 0.0005 < *p* < 0.005, *** indicates *p* < 0.0005. HGRE, high-gray-run emphasis; HGZE, high-gray zone-run emphasis; SRHGRE, Short-run high-gray-run emphasis; SZHGE, short-zone high-gray-run emphasis.

While the relationship between ΔTextures and pathologic response generally became stronger with the increase in the number of discrete values, ΔRLM-derived textures and ΔHigh-ray-zone emphasis significantly distinguished non-responders from partial and complete responders for all discrete values (AUC = 0.70–0.77, *p* < 0.02) (Figure 3). Although ΔShort-zone high-gray emphasis significantly differentiated between complete and non-responders (AUC = 0.69–0.75, *p* < 0.05) for over 128 discrete values, the differentiation was poor for the texture computed with PET images resampled to <128 values with AUC~0.55 (*p* > 0.55). ΔEntropy computed with 32–256 discrete values increased its performance and significance between AUC = 0.55–0.79 and 0.59–0.71 for complete/non-responders and partial/non-responders differentiations respectively as observed in Figure 3.

The MTV_30%_, MTV_40%_, MTV_50%_, and MTV_60%_ on pretreatment PET images had median value of 28, 19, 12, and 7 cm^3^, respectively. The median posttreatment MTV_30%_, MTV_40%_, MTV_50%_, and MTV_60%_ was 26, 14, 8, and 4 cm^3^, respectively.

**Figure 4:**
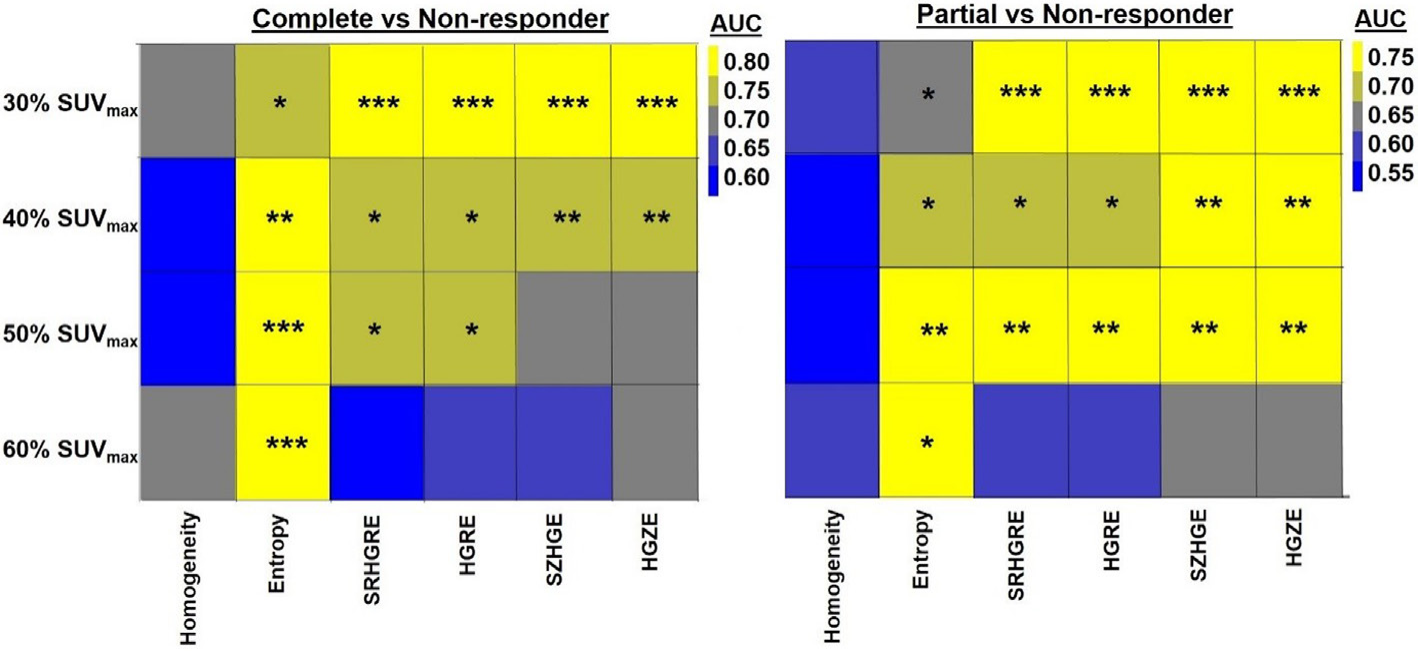
**Heatmap shows the quantification (AUC) of the relationship between ΔTexture and pathologic response with the metabolic tumor volumes (MTV) of >30, 40, 50, and 60% SUV_max_**. * indicates 0.005 < *p* < 0.05, ** indicates 0.0005 < *p* < 0.005, *** indicates *p* < 0.0005. HGRE, high-gray-run emphasis; HGZE, high-gray zone-run emphasis; SRHGRE, Short-run high-gray-run emphasis; SZHGE, short-zone high-gray-run emphasis.

While the relationship between the ΔTextures and pathologic response became stronger with decrease in threshold values, significant differentiation between complete and non-responders was found with ΔEntropy for all threshold values with AUC = 0.73–0.80, *p* < 0.05 (Figure 4). Figure 4 also shows that the ΔRLM textures computed with threshold values of 30–50% SUV_max_ both led to significant differentiation of non-responders from complete and partial responders with AUC = 0.71–0.81 (*p* < 0.02). ΔTextures computed within MTV_60%_ were least related to the pathologic response. Response differentiation based on the 60% SUV_max_ volume calculated ΔRLM were moderate with only with AUC ~0.60 (*p* > 0.30) (Figure 4).

None of the threshold value, discrete value, and texture combination significantly differentiated complete from partial responders with AUC = 0.50–0.65 (*p* > 0.15). Regardless of the resampling schemes and threshold values, among all the textures, Homogeneity had the worst performance in identifying non-responders from complete (AUC = 0.51–0.66) and partial responders (AUC = 0.50–0.59) with *p* > 0.20.

### Survival Analysis

**Figure 5:**
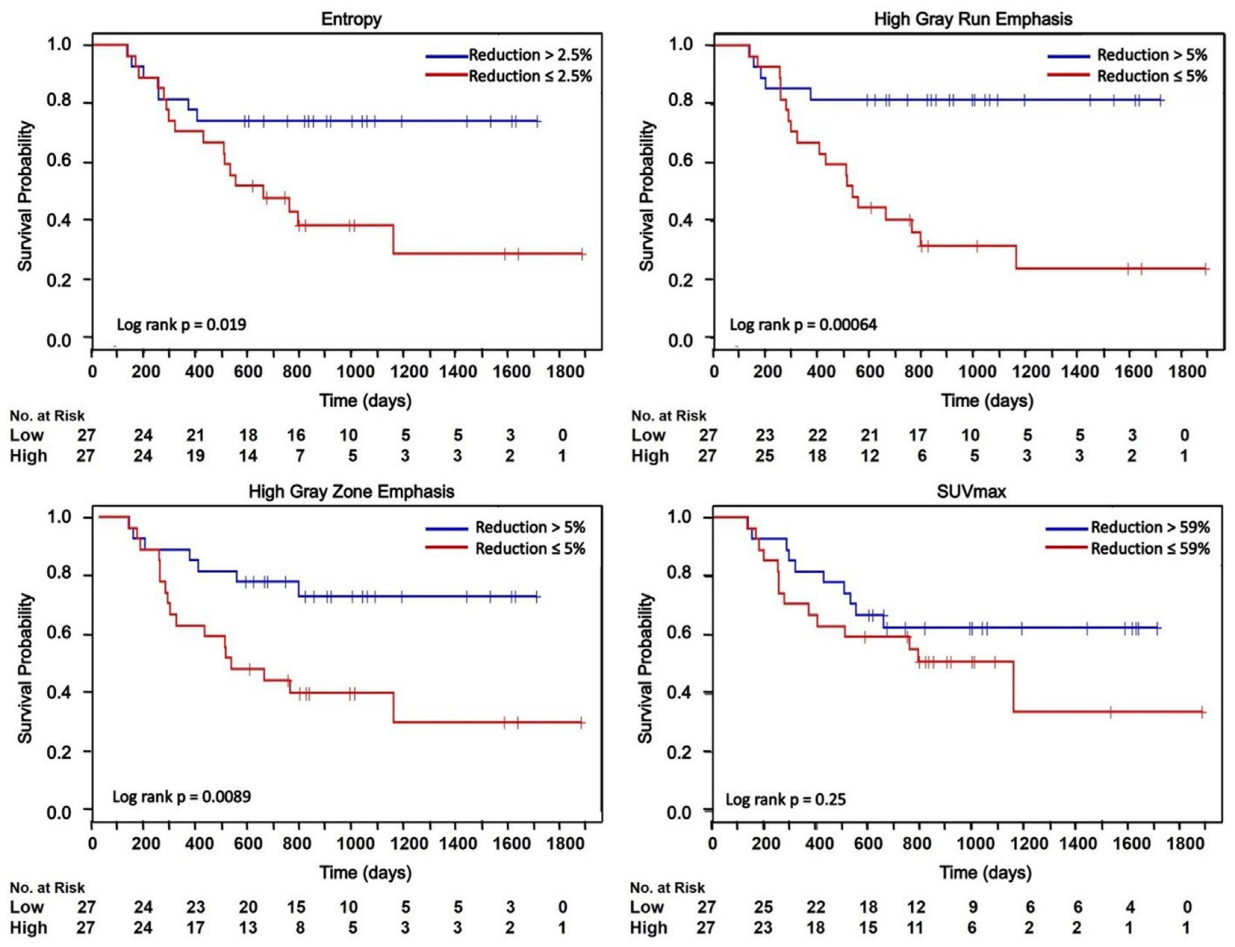
**Kaplan–Meier curves dichotomized based on the median of ΔTextures and ΔSUV_max_**. HGRE, high-gray-run emphasis; HGZE, high-gray zone-run emphasis; SRHGRE, Short-run high-gray-run emphasis; SZHGE, short-zone high-gray-run emphasis. + indicates censored data.

The overall survival was defined as the time from initiation of treatment to patient’s death or censoring time. The median follow up of all 54 patients was 24.7 months. The median survival was 25.5 months. Kaplan–Meier curves shown in Figure 5. Figure 5 demonstrated that median ΔEntropy, ΔHigh-gray-run emphasis, ΔShort-run high-gray-run emphasis, and ΔHigh-gray-zone emphasis significantly discriminated patients with poor and good survival (log-rank test *p* < 0.02). Median values of Short-zone high-gray emphasis, ΔHomogeneity, ΔSUV_max_, and ΔSUV_mean_ failed to stratify patients into different survival groups (log-rank test *p* = 0.25–0.68).

However, *c*-index indicated that the performance of ΔHigh-gray-run emphasis, ΔShort-run high-gray-run emphasis, and ΔHigh-gray-zone emphasis were moderately related to the patients’ overall survival (*c*-index = 0.61–0.62, *p* = 0.06–0.08). All other measures performed poorly related to the overall survival (*c*-index = 0.52–0.58, *p* > 0.22).

## Discussion

Although changes in SUV measures and PET-based textural features during treatment have shown promise in tumor response prediction, it is unclear which quantitative measure is the most predictive. In this study, we attempted to generate a hypothesis regarding which texture features, if any, should be explored as predictors of pathologic response and patient outcome.

Temporal changes in textural features are significantly related to the pathologic response to preoperative chemoradiotherapy, whereas SUV measures are not. Weber et al. observed the change in tumor [^18^F]FDG-PET uptake 2 weeks after neoadjuvant chemotherapy in 40 esophageal cancer patients. They found that the reduction of tumor SUV_max_ by 35% can best predict pathologic response with over 90% sensitivity and specificity (21). Song et al. found that the decrease in average tumor metabolic activity (SUV_mean_) significantly correlated with the pathologic response in 32 esophageal cancer patients undergoing neoadjuvant chemoradiotherapy (57). However, many studies, including ours, fail to confirm the association between the SUV measures and pathologic response (58–61). The conflicting findings may suggest that the SUV measures are inadequate for tumor characterization as they cannot fully describe the heterogeneity of intratumoral [^18^F]FDG distribution (25, 26). Studies therefore have proposed to use imaging features extracted from PET images to describe the [^18^F]FDG uptake heterogeneity (25, 26). Accurate description of the heterogeneous [^18^F]FDG distribution is important for assessing the underlying spatial variation in tumor biological and genetic properties (24), which may provide valuable information to improve treatment outcome prediction (22, 39). Our study confirms this hypothesis and finds that the changes in local GLCM-Entropy and regional (run length and SZM) textures (AUC >0.70) between longitudinal PET images outperformed the SUV measures (AUC ~0.55) in differentiating non-responders from complete and partial responders.

Computation of textural features requires a resampling scheme with at least 128 discrete values and MTV threshold value no more than 40% SUV_max_. Orlhac et al. computed 31 PET-based textures using resampling schemes with 8 to 128 discrete values in 188 lesions from metastatic colorectal, lung, and breast cancer patients (62). They showed that the textures, especially Entropy and Short-zone high-gray emphasis, computed with <32 values are unreliable. They thus concluded that the textures should be computed with at least 32 discrete values. We also observed that the relationship between ΔTextures and pathologic response became stronger with the number of discrete values in the resampling schemes (Figure 3). Particularly, ΔEntropy and ΔShort-zone high-gray emphasis were found to be least robust to the resampling schemes (Figure 3). ΔEntropy and Short-zone high-gray emphasis computed with 32 and 64 discrete values performed poorly in separating complete and non-responders (AUC <0.60, *p* > 0.37), while the performance improved when 128 and 256 discrete values were used (AUC >0.70, *p* < 0.05) (Figure 3). ΔRLM textures and ΔHigh-gray-zone emphasis are robust to resampling scheme with AUC >0.70 for all discrete values.

Furthermore, we found that the relationship between the pathologic response and ΔTextures, except Entropy, became weaker with the increase in metabolic volume threshold values. Hatt et al. computed two local and two regional textures on 555 PET images consisting of breast, cervical, lung, esophageal, and head-and-neck tumors (63). They found that the PET-based textures computed for tumor size <10 cm^3^ do not provide important prognostic information. Our results are consistent with the findings of Hatt et al. We observed in Figure 4 that the ΔTextures computed with tumor volumes <10 cm^3^ based on 50–60% SUV_max_ thresholds were generally less related to the pathologic response than volumes >10 cm^3^ computed with thresholds of 30–40% SUV_max_.

Temporal changes in tumor [^18^F]FDG distribution after chemoradiotherapy assessed by ΔRLM textures were moderately related to the patients’ overall survival. In the survival analysis, we dichotomized the Kaplan–Meier curves according to the median reduction in the texture values. Patients with reduction in texture greater than the median values were found to have significant survival benefit (Figure 5). For example, log-rank test showed that median ΔHigh-gray-run emphasis can significantly discriminate patients with good and poor survivals with *p*-value <10^−3^. However, the results of the Kaplan–Meier curves include a dichotomization based on a *post hoc* cutoff value. Concordance index is a more conservative measure that assesses the relationship between ΔTextures and survival without relying on a particular cutoff value (54). Among all textures, the relationship between the survival and ΔRLM textures was found to be the strongest with *c*-index = 0.62 comparing to the *c*-index <0.55 for ΔSUV_max_ and ΔSUV_mean_. In this study, the textures were extracted from PET images acquired before and after chemoradiotherapy, but prior to the surgery. Incorporating the survival benefit of surgery may lead to improvement of c-index. In future studies, we will build a multivariate predictive model of survival by incorporating the effect of surgery and combined textural features on a larger dataset.

Texture quantification has been shown to be sensitive to the acquisition modes and reconstruction parameters of PET images (64). In this study, we found that the temporal change in textures, such as Run length and Size zone textures, can significantly differentiate pathologic non-responders from responders with AUC >0.70 (*p* < 0.01) (Figure 2), despite the PET images were acquired from five different PET/CT scanners and reconstructed using different reconstruction parameters. We showed using Kruskal–Wallis test that the differences in ΔSUV_max_ and ΔSUV_mean_ between different PET/CT scanners were not significant (*p* = 0.651 and *p* = 0.287 for ΔSUV_max_ and ΔSUV_mean_, respectively) (results not shown). The SUV measures were observed to decrease the most in images acquired by the GE discovery RX scanner. In particular, the average ΔSUV_max_ was found to be −53.5 ± 20.4, −55.4 ± 33.0, −64.0 ± 24.5, −47.9 (only one patient), and −57.1 ± 33.3 for the GE Discovery ST, STE, RX, and LS, and Siemens Biograph PET/CT scanners, respectively. The average ΔSUV_mean_ was found to be −69.7 ± 21.3, −79.0 ± 17.2, −85.1 ± 8.2, −67.58 (only one patient), and −73.8 ± 29.7 for the GE Discovery ST, STE, RX, and LS, and Siemens Biograph PET/CT scanners, respectively. Our results suggest that some textures may be robust to PET reconstruction parameters in identifying pathologic responders. However, this hypothesis needs to be further investigated.

## Conclusion

The temporal changes in all textures, except Homogeneity, were better correlated to pathologic response and overall survival than the SUV_max_ and SUV_mean_. Computation of the PET-based textural features requires a resampling scheme with at least 128 discrete values and MTV threshold value no more than 40% SUV_max_. The hypothesis that the temporal changes in PET-based textures can be used as clinical predictors of better patient outcomes will be tested in a larger patient dataset in the future.

## Author Contributions

SY, NS, HM, HA, and RB conceived and designed the study. SY and TC performed the data analysis. NS and HM contributed data. HA and TC provide expert guidance and contributed analysis tools. SY and RB wrote the paper. TC, NS, HM, and HA reviewed the manuscript.

## Conflict of Interest Statement

The authors declare that the research was conducted in the absence of any commercial or financial relationships that could be construed as a potential conflict of interest.
